# Long non-coding RNA HOTTIP exerts an oncogenic function by regulating HOXA13 in nasopharyngeal carcinoma

**DOI:** 10.1007/s11033-023-08598-9

**Published:** 2023-07-01

**Authors:** Huajun Feng, Feipeng Zhao, Jian Luo, Shengen Xu, Zhuoping Liang, Wei Xu, Yilin Bao, Gang Qin

**Affiliations:** Department of Otolaryngology Head and Neck Surgery, the Affiliated Hospital of Southwest Medical University, No.25, Taiping Street, Jiangyang District, Luzhou, 646000 Sichuan China

**Keywords:** Nasopharyngeal carcinoma, Long noncoding RNA, HOTTIP, HOXA13, Oncogenic

## Abstract

**Background:**

The long non-coding RNA HOXA transcript at the distal tip (HOTTIP) and homeobox A13 (HOXA13) have been identified as oncogenes that play a pivotal role in tumorigenesis. However, their specific mechanisms of action in nasopharyngeal carcinoma (NPC) progression remain unclear.

**Methods and results:**

In the present study, RT-qPCR was employed to quantify RNA expression in NPC cells and tissues. Flow cytometry, MTT, CCK8 and colony formation assays were utilized to assess cell apoptosis and proliferation. Transwell assay was conducted to evaluate migration and invasion while Western blotting was performed for protein expression analysis. Our findings revealed that the expression of HOTTIP was significantly upregulated in NPC cell lines. Inhibition of HOTTIP could induce apoptosis and suppress proliferation, clonogenicity, invasion and metastasis in NPC cells. Knockdown of HOTTIP led to downregulation of HOXA13 expression, which subsequently inhibited the proliferation and metastasis in NPC cells. The inhibitory effects on cell proliferation and metastasis caused by HOTTIP silencing were rescued by HOXA13 overexpression. Additionally, there was a significant positive correlation between HOTTIP and HOXA13, which were found to be elevated in NPC tissues compared to normal tissues.

**Conclusions:**

We have determined that LncRNA HOTTIP facilitates tumorigenesis by modulating the expression of HOXA13 in NPC cells. Targeting HOTTIP/HOXA13 may be a promising therapeutic strategy for NPC.

**Supplementary Information:**

The online version contains supplementary material available at 10.1007/s11033-023-08598-9.

## Introduction

Nasopharyngeal carcinoma (NPC), one of the most common head and neck tumor, is prevalent in Southeast Asia, especially in Southern China [1, 2]. Radiotherapy in combination with chemotherapy is employed as the primary treatment for NPC [3]. Although intensity-modulated radiation therapy and chemoradiotherapy do work well, the result is still disappointing [4]. NPC has great invasion and metastasis potential [5]. Therefore, we need to further understand the molecular mechanisms associated with NPC to develop novel treatment.

Progresses in molecular biology identified new types of long non-coding RNAs (lncRNAs) and their specific functions in eukaryotic cells [6]. Many studies highlighted that lncRNAs are involved in diverse biological processes of cancer, such as cell growth, metastasis, apoptosis, development and progression of cancer [7]. In recent years, growing evidence has substantiated the pivotal role of lncRNA in nasopharyngeal carcinoma. LncRNAs such as SNHG16, LINC00173, CASC19, OIP5-AS1 and others have been implicated in the progression of NPC and its resistance to radiotherapy [8–11]. Furthermore, lncRNAs may serve as potential biomarkers for predicting metastasis in NPC patients [12].

LncRNA HOTTIP is located at the 5’ end of the homeobox A (HOXA) cluster and controls the activation of several 5’ HOXA genes [13]. HOTTIP have been shown to be involved in many biological processes, such as cell growth, cell migration, cell invasion, cell apoptosis [14, 15]. Several reports documented the modulation of HOTTIP expression in a variety of tumors, such as hepatocellular carcinomas, colorectal cancer, gastric cancer, pancreatic cancer as well as osteosarcoma and thereby of great value for diagnostic screening and therapeutic intervention [16–20]. Recent study demonstrated that HOTTIP promoted NPC cell proliferation, migration and invasion by inhibiting miR-4301 [21]. Nonetheless, the mechanism of lncRNA in NPC still needs to be further explored.

As the most posterior of the homeobox genes, the homeobox A13 (HOXA13) is shown to regulate mammalian embryonic development and participate in tumorigenesis [22, 23]. Several documents illustrated the aberrant expression of HOXA13 in a variety of tumors, such as gastric cancer, hepatocellular carcinomas and prostatic neoplasia [24–26]. Han Y and colleagues demonstrated that HOXA13 expression was elevated in cancerous tissues compared with the corresponding non-cancerous mucosa and expression of HOXA13 was associated with aggressive phenotype of gastric cancer [27]. These studies indicated that HOXA13 could be a prognostic marker for certain cancer. Some studies suggested that HOTTIP functions as an oncogene by regulating HOXA13 expression in esophageal squamous carcinoma and gastric cancer [28, 29]. However, the relationship between HOTTIP and HOXA13 in NPC is unclear.

The HOTTIP gene is located in physical contiguity with HOXA13 and directly controls the HOXA locus gene expression by way of interaction with the WDR5/MLL complex. Based on these studies, this research focused on how HOTTIP and HOXA13 influence the progression of NPC. We hope that the results of our study will provide a reference for the treatment of NPC.

## Materials and methods

### Cell culture and lentiviral-mediated shRNA transduction

An immortalized nasopharyngeal epithelial cell line NP69 was cultured in Keratinocyte-SFM (Gibco; Life Technologies, CA, USA) and human NPC cell lines CNE1, CNE2, HNE-1 and 5-8F were cultured in RPMI-1640 (Gibco; Life Technologies, CA, USA). All cells were purchased from central south university, Hunan Province, China. Cells were supplemented with 10% fetal bovine serum (FBS; Biological Industries, Israel), 100 µg/ml penicillin, 0.1 mg/ml streptomycin and 2 mm L-glutamine. Cells were cultured at 37°C in a humidified atmosphere containing 5% CO_2_. To construct a lentiviral vector expressing shRNA against HOTTIP, we used the pLKO.1puro-vector ligated with 58 base pair-oligos. The HOTTIP shRNA forward is 5’-CCGGGGAAGAAATTCAATGCCATGCCTCGAGGCATGGCATTGAATTTCTTCCTTTTTG-3’ and the reverse is 5’-AATTCAAAAAGGAAGAAATTCAATGCCATGCCTCGAGGCATGGCATTGAATTTCTTCC-3’. In short, lentiviral particles were produced by transfecting 293 FT cells, viral supernatants were collected 48 h after transfection. Cells were transduced with virus-containing medium in the presence of 4 ng/ml Polybrene (Zhong Qiao Xin Zhou Biotechnology Co.,Ltd, Shanghai, China) and then were selectively isolated with 2 µg/ml puromycin ((InvivoGen, San Diego, CA, USA), and pooled for the next assays. The use of all cells was approved by the ethics committee of the Affiliated Hospital of Southwest Medical University.

### Tissue specimens

Human nasopharyngeal carcinoma specimens were collected from patients undergoing surgical resection in the Affiliated Hospital of Southwest Medical University. The specimens were confirmed as nasopharyngeal carcinoma by pathological analysis. Fresh nasopharyngeal carcinomas and normal tissues were immediately snap-frozen in liquid nitrogen and preserved in -80 °C until use. All specimens were evaluated, reclassified according to current WHO and UICC-TNM classification (Travis et al., 2004) (7th edition; Sobin et al., 2009). This study was approved by the Institutional Review Board of our hospital (K2016050), and written informed consent about analysis of their clinical parameters was obtained from individual patients before treatment.

### Plasmid construction

The HOXA13 overexpression construct was based on the piRES2-EGFP vector. The DNA fragment of HOXA13 (GenBank accession No. NM_000522.4) was obtained by PCR using the forward primer F: 5’-TCAAGCTTCGAATTCATGACAGCCTCCGTGCTCC-3’, and reverse primer R: 5’-GAGAGGGGCGGATCCTTAACTAGTGGTTTTCAGTTTGTTGATGAC-3’, and was cloned into the EcoRI/Bam HIsites.

### RNA interference

The stealth RNAi oligonucleotides specifically targeting the HOTTIP and HOXA13 were synthesized by company (Ribobio, Guangzhou, China). HOTTIP-siRNA1, sense: 5’ GCCUCAUCGAGAAGAAUAUTT 3’, antisense: 5’ AUAUUCUUCUCGAUGAGGCTT 3’. HOTTIP-siRNA2, sense: 5’ GGGACUGUGUCUCUGUUAUTT 3’, antisense: 5’ AUAACAGAGACACAGUCCCTT 3’. HOTTIP-siRNA3, sense: 5’ GCCUCAUCGAGAAGAAUAUTT 3’, antisense: 5’ AUAUUCUUCUCGAUGAGGCTT 3’. HOXA13-siRNA1, sense: 5’ GCCUCAUCGAGAAGAAUAUTT 3’, antisense: 5’ AUAUUCUUCUCGAUGAGGCTT 3’. HOXA13-siRNA2, sense: 5’ GGGACUGUGUCUCUGUUAUTT 3’, antisense: 5’ AUAACAGAGACACAGUCCCTT 3’. HOXA13-siRNA3, sense: 5’ GCCUCAUCGAGAAGAAUAUTT 3’, antisense: 5’ AUAUUCUUCUCGAUGAGGCTT 3’.

### Total RNA extraction and quantitative real-time PCR

Total RNA of cells and tissues were purified with TRIzol reagent (Invitrogen, San Diego, CA, USA) according to the manufacturer’s protocol and measured with a NanoDrop ND-1000 spectrophotometer (Thermo Fisher Scientific, CHI, USA). Then, equal amounts of total RNA (1 µg) were reverse-transcribed with the PrimerScriptRT reagent kit with gDNA Eraser (TaKaRa, Japan). The levels of genes were amplified with the ABI PRISM 7500 Fast Real-Time PCR System (Applied Biosystems, USA) and the SYBR Green array (TaKaRa, Japan). GAPDH was measured as control and arbitrary units are used to display the normalized relative gene expression. Primers for HOTTIP in qRT-PCR experiments are the forward primer F: 5’-CCTAAAGCCACGCTTCTTTG-3’, and reverse primer R: 5’-TGCAGGCTGGAGATCCTACT-3’. Primers for HOXA13 are the forward primer F: 5’-CGCTTCAGAACTCGTTGCTTTGC-3’, and reverse primer R: 5’- CGGAAGAACTGGCAG-TCTTTACCT-3’. Primers for GAPDH are the forward primer F: 5’-GGACTTCGAGCAAGAGATGG-3’, and reverse primer R: 5’-AGCACTGT GTTGGCGTACAG-3’.

### Western blot

Cells and tissues were lysed using RIPA buffer (1% NP-40, 0.1% SDS, 0.5% DOC, 150 mM NaCl, 10 mM Tris-HCl, and protease inhibitor) and then the cell lysis was used for Western blot as described previously [30]. Rabbit polyclonal anti- HOXA13 antibody (ab106503, 1:200), mouse monoclonal anti-GAPDH antibody (ab8245, 1:750), rabbit anti-beta Actin antibody (ab8227, 1:5000) were purchased from Abcam (Cambridge, UK). Immunoreactivity was visualized with a chemiluminescence detection kit (Donghuan Biotech, Shanghai, China).

### Flow cytometric analysis

Cell apoptosis was analyzed by flow cytometry with annexin V-FITC/propidium iodide (PI) apoptosis detection kit (BD Biosciences, San Jose, CA, USA). Briefly, cells were fostered for 48 h in 6-well plates at 3 × 10^5^ cells/well. Then, cells were collected and washed twice by PBS (PH = 7.4), and resuspended in 100 µl of binding buffer. After that, cells were incubated for 10 min with 5 µl of PI and 10 µl of annexin V-FITC in the dark.

### Cell viability assay

For MTT, briefly, cells were planted in 96-well plates (Corning, NY, USA) at a density of 1 × 10^3^ cells and added 20 µl of MTT(Solarbio, Beijing, China) into each well 0 h, 24 h, 48 h,72 h, 96 h later according to the manufacturer’s protocol. For 4 h later, the medium was removed and 150 ml DMSO was added to each well. After 10 min of vibration mixing, the optical density (OD) was measured at 490 nm with a microplate reader. CCK8 assay was carried out using the protocol described previously [31]. Briefly, cell was planted in 96-well plates (Corning, NY, USA) at a density of 3 × 10^3^ cells and added 10 µl of CCK8 into each well 24 h later according to the manufacturer’s protocol. Four 24 h later, cell viability was determined by measuring the absorbance of the converted dye at 450 nm.

### Colony formation assay

Cells were plated at a density of 200 cells/well in 6-well plates, and cultured for 1–2 weeks. The medium was changed timely. When most visible colonies had expanded with more than 50 cells, colonies were fixed immediately in 4% paraformaldehyde for 30 min, and stained with crystal violet for 30 min. After the dye was washed out, the colonies were manually counted and plates were photographed.

### Cell migration and invasion assays

Transwell migration and invasion assay was performed with Transwell (Millipore, MA, USA) according to the manufacturer’s protocol. Briefly, after different treatment, cells were planted into the top chambers of transwell plates coated with collagen IV or not, and medium without cells was placed to the bottom chambers. After incubation about 24 h at 37 °C, cells were stained with 0.1% crystal violet at the room temperature for 25 min. Then, cells on top of each filter were removed and migrating cells were counted.

### Statistical analysis

SPSS 19.0 statistical analysis software was used to analyze these experimental results. Data are expressed as mean ± standard deviation (S.D.). The statistical significance of differences between two groups was analyzed with a paired two-tailed Student’s t-test and between multiple groups was analyzed with oneway ANOVA. The symbol * denotes statistical difference (*P* < 0.05), while ** represents great significant difference (*P* < 0.01) in a two-tailed Student’s t-test.

## Results

### The expression of HOTTIP was upregulated in NPC cell lines

**Fig. 1:**
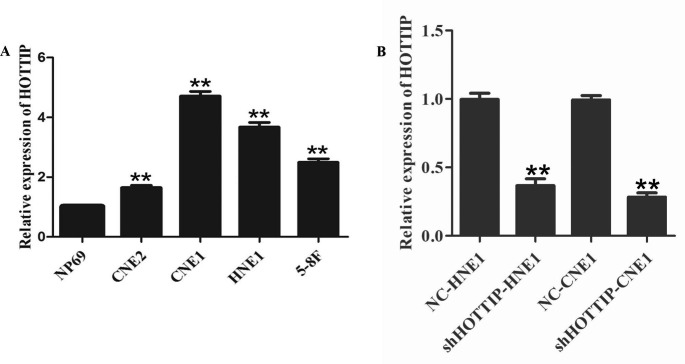
The expression of HOTTIP is upregulated in NPC cells. (**A**) RT-qPCR analysis on four NPC cell lines including CNE2, CNE1, HNE1, 5-8 F and one immortalized nasopharyngeal epithelial cell line NP69. Relative to non-tumorous cell NP69, the NPC cell lines showed higher expression levels of HOTTIP RNA, especially in CNE1 and HNE1. **P < 0.01 vs.NP69. (**B**) RT-qPCR demonstrated that shHOTTIP could repress the expression of HOTTIP effectively in the CNE1 and HNE1 cells. **P < 0.01 vs. control. NC: negative control group (cells transfected with an ineffective HOTTIP interference sequence)

To investigate the expression of HOTTIP at transcriptional level in NPC cell lines, we first performed the RT-qPCR analysis on four NPC cell lines including CNE2, CNE1, HNE1 and 5-8 F and one immortalized nasopharyngeal epithelial cell line NP69. Relative to non-tumorous cell, the NPC cell lines showed HOTTIP was upregulated, especially in CNE1 and HNE1 (Fig. 1A). Therefore, we concluded that the expression of HOTTIP was upregulated in NPC cell lines.

### Repression of HOTTIP promoted apoptosis and inhibited proliferation, clonogenicity of NPC cells

**Fig. 2:**
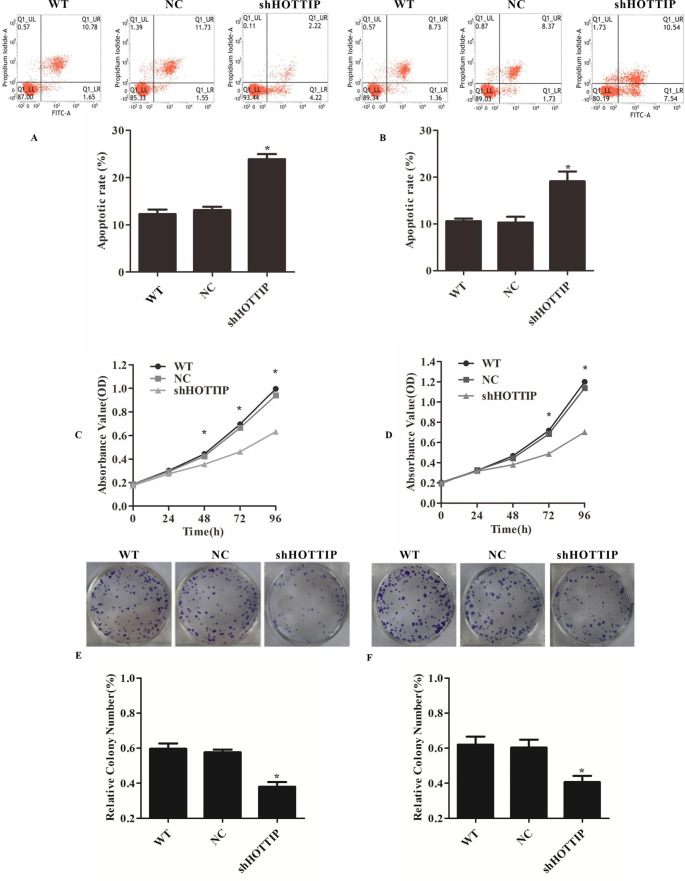
Repression of HOTTIP promoted apoptosis and inhibited proliferation, clonogenicity of NPC cells. (**A**) and (**B**) Flow cytometry analysis revealed that HOTTIP knockdown promoted the apoptosis of CNE1 and HNE1 cells. (**C**) and (**D**) MTT assay analysis revealed that HOTTIP knockdown repressed the proliferation of CNE1 and HNE1 cells. (**E**) and (**F**) Colony formation analysis revealed that HOTTIP knockdown repressed the colony formation ability of CNE1 and HNE1 cells. *P < 0.05 vs. control. **P < 0.01 vs. control. NC: negative control group (cells transfected with an ineffective HOTTIP interference sequence).WT: Wild type of CNE1 and HNE1 cells

To elucidate the possible role of HOTTIP in NPC, we first designed shRNA against HOTTIP (shHOTTIP) and transfected CNE1 and HNE1 cells with shRNA. The results demonstrated that shHOTTIP could effectively repress the expression of HOTTIP (Fig. 1B). Then, we investigated the function of HOTTIP in NPC cell lines transfected with shHOTTIP. Flow cytometry analysis was demonstrated that HOTTIP knockdown promoted cell apoptosis of CNE1 and HNE1 cells (Fig. 2A and B). MTT and colony formation analysis showed that HOTTIP knockdown repressed cell proliferation and colony formation ability of CNE1 and HNE1 cells (Fig. 2C-F). These results showed that HOTTIP played a growth-promoting role in NPC cells.

### Repression of HOTTIP inhibited cell migration and invasion of NPC cells

**Fig. 3:**
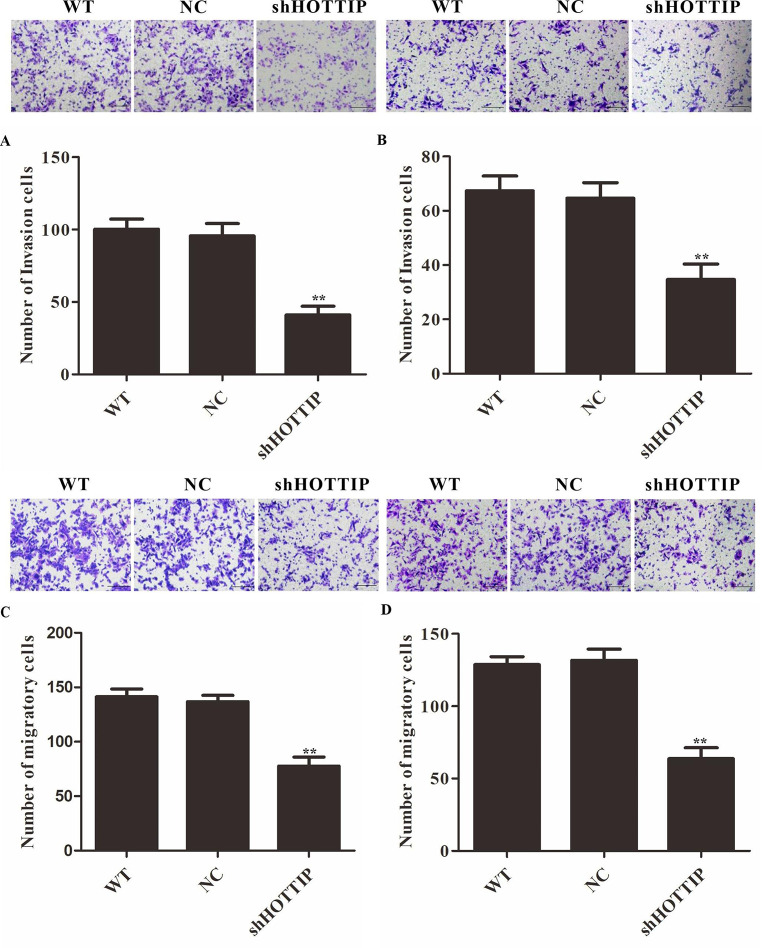
Repression of HOTTIP inhibited cell migration and invasion of NPC cells. (**A**) and (**B**) HOTTIP knockdown inhibited the invasion of CNE1 and HNE1 cells. (**C**) and (**D**) HOTTIP knockdown inhibited the migration of CNE1 and HNE1 cells. Scale bar = 50 μm. **P < 0.01 vs. control. NC: negative control group (cells transfected with an ineffective HOTTIP interference sequence). WT: Wild type of CNE1 and HNE1 cells

Furthermore, we investigated the effect of HOTTIP on the migration and invasion of CNE1 and HNE1 cells. The results showed that knockdown of HOTTIP could inhibit cell invasion and migration of CNE1 and HNE1 cells (Fig. 3A-D). Taken together, these results indicated that HOTTIP promoted tumorigenesis in NPC cells.

### Knockdown of HOTTIP repressed HOXA13 expression in NPC cells

**Fig. 4:**
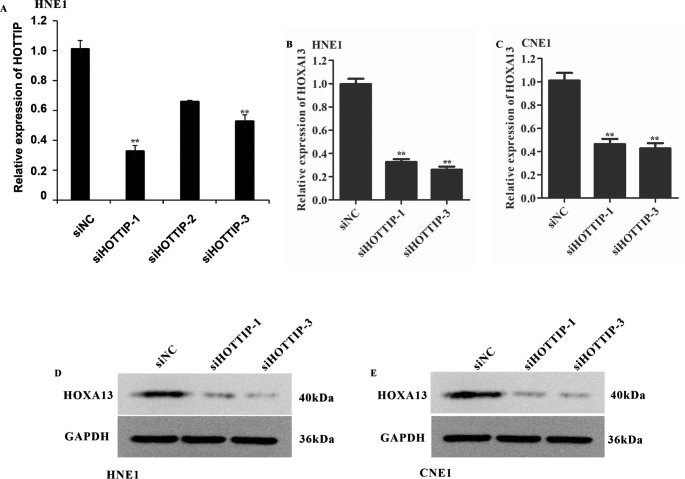
Inhibition of HOTTIP expression decreased the HOXA13 expression in NPC cells. (**A**) RT-qPCR analysis of the HOTTIP expression in HNE1 cells transfected with siRNAs. **P < 0.01 vs. siNC. (**B**) and (**C**) RT-qPCR analysis of the HOXA13 expression in HNE1 and CNE1 cells transfected with siHOTTIP and control siRNAs. **P < 0.01 vs. siNC. (**D**) and (**E**) Western blot analysis of the HOXA13 expression in HNE1 and CNE1 cells transfected with siHOTTIP and control siRNAs. NC: negative control group (cells transfected with an ineffective HOTTIP interference sequence)

Previous studies documented that HOTTIP could regulate HOXA13 expression and affect tumorigenesis. Firstly, we designed three siRNAs against HOTTIP and transfected HNE1 cells with these siRNAs. The results demonstrated that all the three HOTTIP siRNAs, especially siHOTTIP-1 and siHOTTIP-3 could repress the expression of HOTTIP (Fig. 4A). To determine whether HOTTIP could regulate HOXA13 expression in NPC cell lines, we detected HOXA13 expression in CNE1 and HNE1 cells transfected with siHOTTIP-1 and siHOTTIP-3. The results demonstrated that the inhibition of HOTTIP could repress HOXA13 mRNA level in HNE1 and CNE1 cells (Fig. 4B and C). Furthermore, the protein level of HOXA13 was also decreased in HNE1 and CNE1 cells with HOTTIP knockdown (Fig. 4D and E). Taken together, these results indicated that the expression of HOXA13 can be regulated by HOTTIP in human NPC cells.

### Repression of HOXA13 inhibited the proliferation and metastasis of HNE1 cells

**Fig. 5:**
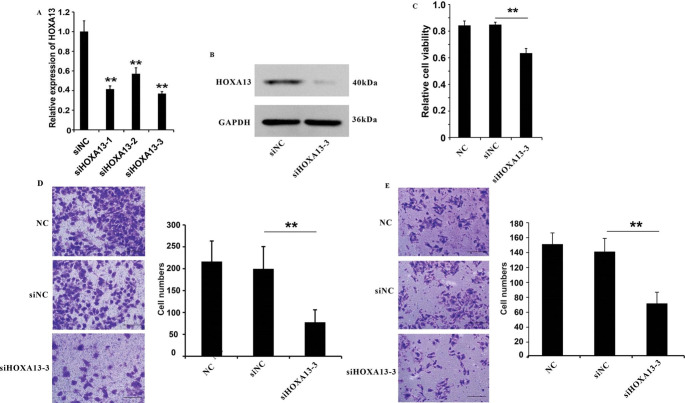
Knock-down of HOXA13 inhibit the proliferation and metastasis of HNE1 cells. (**A**) RT-qPCR analysis of the efficiency of siHOXA13 in HNE1. (**B**) Western blot analysis of the efficiency of siHOXA13 in HNE1 cells transfected with siRNA against HOXA13 and control siRNAs. (**C**) CCK8 assay analysis of cell proliferation in HNE1 cells transfected with siHOXA13 or control siRNA. (**D**) Cell migration of siHOXA13 HNE1 cells and control cells. Scale bar = 25 μm. (**E**) Cell invasion assay analysis of cell invasion in HNE1 cells transfected with siHOXA13 or control siRNA. Scale bar = 25 μm. **P < 0.01. vs. siNC. NC: negative control group (cells transfected with an ineffective HOXA13 interference sequence)

HOTTIP could affect the growth and metastasis of NPC cells, and regulated the expression of HOXA13, we believed that HOTTIP might play its role by regulating the expression of HOXA13 in NPC cells. To test this hypothesis, we firstly dissected the function of HOXA13 in HNE1 cells. We designed siRNAs specific to HOXA13 and transfected HNE1 cells with these siRNAs. The results showed that the siHOXA13-3 siRNA could repress the HOXA13 expression more than siHOXA13-1 and siHOXA13-2 (Fig. 5A). Western blot analysis showed that the siHOXA13-3 siRNA could repress the HOXA13 expression in protein level (Fig. 5B). Interestingly, we found that knockdown of HOXA13 inhibited the cell growth in CCK8 assay (Fig. 5C). Moreover, transwell assay demonstrated that repression of HOXA13 inhibited cell migration and invasion of HNE1 cells (Fig. 5D and E). In summary, these results illustrated that HOXA13 could affect the cell growth, migration and invasion of HNE1 cells.

### Modulation of HOXA13 expression affected the function of HOTTIP in NPC cell lines

**Fig. 6:**
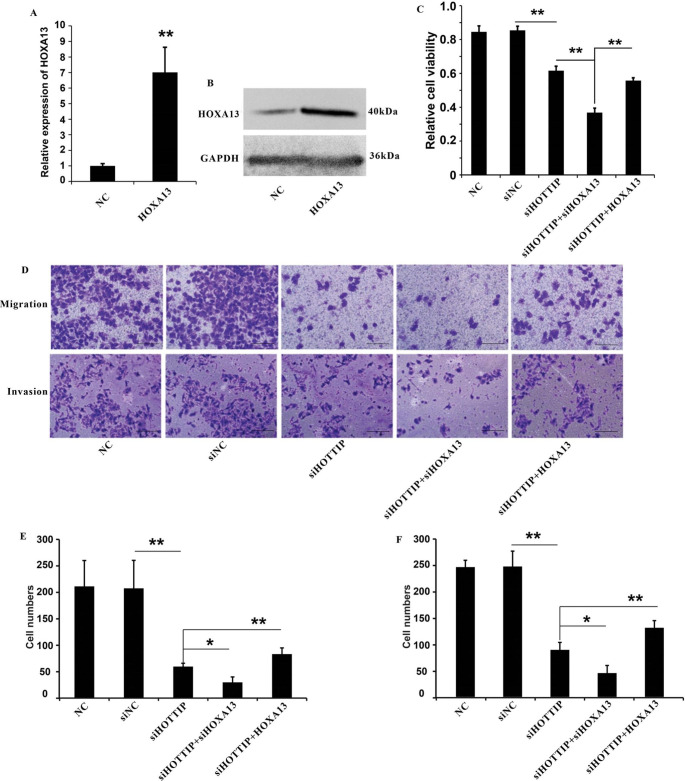
Modulation of HOXA13 expression could affect the function of HOTTIP in HNE1 cells. (**A**) RT-qPCR analysis of the overexpression of HOXA13. (**B**) Western blot analysis of the overexpression of HOXA13. (**C**) CCK8 assay analysis of cell proliferation in HNE1 cells. (**D**) Cell migration and cell invasion assay analysis of cell metastasis in HNE1 cells. Scale bar = 25 μm. (**E**) Statistic analysis of cell migration assay. (**F**) Statistic analysis of cell invasion assay. GAPDH expression was measured as control. *P < 0.05, **P < 0.01. NC: negative control group (cells transfected with an ineffective sequence)

To determine whether HOTTIP promoted cell growth and metastasis through modulating HOXA13 expression, we investigated whether the level of HOXA13 could affect HOTTIP function in HNE1 cells. We overexpressed HOXA13 in HNE1 cells, qRT-qPCR and western blot analysis showed an efficient HOXA13 overexpression in HNE1 cells (Fig. 6A and B). Firstly, we found that HNE1 cell growth was repressed by HOTTIP knockdown and the effect of growth inhibition was even more significant when HOXA13 was knockdown(Fig. 6C). In addition, the inhibition on cell growth was partially restored if HOXA13 was overexpressed (Fig. 6C). The colony formation assay demonstrated that the reduced ability of HNE cells to form colonies resulting from HOTTIP knockdown could be rescued by overexpressing HOXA13. (Supplementary Fig. 1). Furthermore, the inhibition of cell migration and invasion caused by HOTTIP knockdown could be improved by HOXA13 overexpression, and could be enhanced by HOXA13 knockdown (Fig. 6D-F).

### Positive correlation of HOTTIP and HOXA13 expression in NPC

**Fig. 7:**
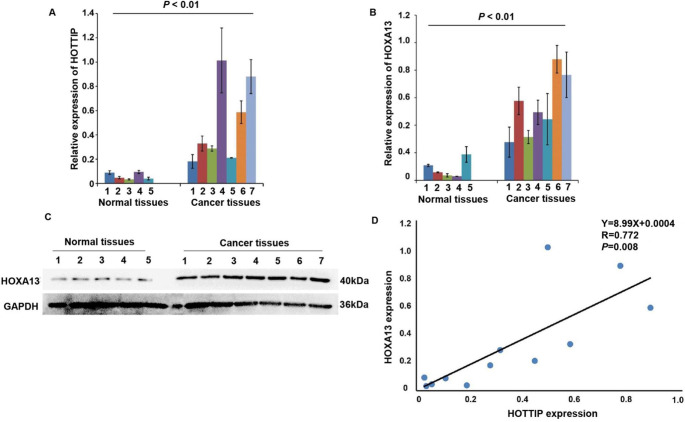
Positive correlation of HOTTIP and HOXA13 expression in normal tissues and NPC tissues. (**A**) RT-qPCR analysis of HOTTIP RNA transcripts in normal and cancer tissues. (**B**) RT-qPCR analysis of HOXA13 mRNA level. (**C**) Western blot analysis of HOXA13 protein level. (**D**) Spearman test was used to analyze correlation of HOTTIP and HOXA13 expression in NPC. *P* < 0.01: two-tailed unpaired student’s t-test

Above all, we have found that HOTTIP and HOXA13 could affect the cell growth and cell metastasis in NPC cell lines. To investigate the potential relationship of HOTTIP and HOXA13 with tumorigenesis in NPC tissues, we compared their expression level in clinical NPC samples and normal tissues by RT-qPCR and Western blot assay. The clinical and pathologic characteristics of NPC and normal patients were shown in Table 1. Student’s *t*test showed that the RNA level of HOTTIP in all the cancer tissues was much higher than that in normal tissues (Fig. 7A). The results analysis demonstrated that both mRNA and protein level of HOXA13 were elevated in cancer tissues, compared with those in normal tissues (Fig. 7B and C). Student’s *t*test also showed a statistically significant difference in the expression level of HOTTIP and HOXA13 between tumor tissues and normal tissues. As expected, the RNA expression level of HOTTIP were positively correlated with HOXA13 in clinical tissues (R = 0.772, *P* = 0.008) (Fig. 7D).

**Table 1:** The clinical and pathologic characteristics of NPC and normal patients

Parameter	NPC patients(N = 7)	normal patients(N = 5)
Age(years)	42.4 ± 4.2	41.3 ± 4.1
Gender(male/female)	3/4	2/3
Pathological type (keratinizing /nonkeratinizing)	0/7	
Clinical stages		
I-II	3	
III-IV	4	
T stage		
T_1 − 2_	3	
T_3 − 4_	4	
N stage		
N_0_	2	
N_1_	4	
N_3_	1	
M stage		
M_0_	7	

## Discussion

LncRNA HOTTIP was reported as an oncogenic gene and plays a key role in tumorigenesis. In this study, we sought to study the function of HOTTIP and HOXA13 in NPC. Firstly, we identified that the expression of HOTTIP was upregulated in NPC cell lines. The shRNA-induced inhibition assay demonstrated that HOTTIP knockdown promoted cell apoptosis and repressed cell proliferation, clonogenicity, migration and invasion of NPC cells. Then, we found that silencing of HOTTIP inhibited HOXA13 expression and HOXA13 exerts a similar function as HOTTIP in NPC. Furthermore, we found that HOXA13 knockdown enhanced the inhibitory effect on cell proliferation and metastasis caused by HOTTIP knockdown, and HOXA13 partially recue this effect, suggesting that HOTTIP promotes tumorigenesis through regulating HOXA13 expression. At last, we found that the HOTTIP and HOXA13 was increased and significantly positively correlated in clinical tissues. In short, we identified the function and mechanism of HOTTIP and HOXA13 in NPC and provided a new way for therapeutic treatment of NPC.

NPC is common head and neck tumors in Southern China. In the last decades, our understanding of physiological and pathological processes of NPC has greatly improved [32, 33]. However, the mechanism of NPC tumorigenesis and progression remains unclear. Emerging evidence demonstrated that lncRNAs affect the cancer progression, such as cell proliferation, apoptosis, and metastasis, differentiation and cycle [34]. Recent advances documented that lncRNAs play important function in NPC, such as lncRNA-ROR, lncRNA-EWSAT1 and so on [35, 36]. Previous studies reported that HOTTIP was up-regulated in a variety of cancers. In this study, we further confirmed that lncRNA HOTTIP plays important function in NPC. We found that siRNA-induced silencing of HOTTIP in HNE1 and CNE2 cell resulted in a significant repression of cell proliferation, migration and invasion. Thus, these results suggested that HOTTIP could exert an oncogenic function in NPC cell lines.

Hox genes, referred to as HoxA, B, C, and D, exert important functions in the establishment of the body architecture [37]. HOXA expression levels was correlate with tumorigenesis in variety of cancers, such as gastric cancer, laryngeal cancer and acute myeloid leukemia [38–40]. HOXA13 was documented as a marker of gut primordial posteriorization and plays a key role in tumorigenesis of variety of cancers. In present study, we demonstrated that siRNA-mediated HOXA13-knockdown inhibited the proliferation, migration and invasion of NPC cells. These studies shown an oncogenic role of HOXA13 in NPC cell lines.

The regulation of HOXA13 by HOTTIP was involved in tumorigenesis [41]. In our study, we also observed siRNA-induced silencing of HOTTIP led to the repression of HOXA13. Further, we found that over-expressed HOXA13 restored the inhibition of cell proliferation, and cell metastasis caused by repressed HOTTIP in NPC cells. Thus, our study illustrated that the regulatory mechanism between HOTTIP and HOXA13 was also conserved in NPC tumorigenesis.

Recently, researchers made great efforts to screen and identify characteristic biomarkers specific for solid tumors. The expression profile of HOTTIP and HOXA13 was documented as prognostic factor for a variety of cancers, such as pancreatic ductal adenocarcinoma and gastric cancer [42, 43]. In our study, we firstly found that the both HOTTIP and HOXA13 exerted oncogenic role in NPC cells. Then we detected the expression of HOTTIP and HOXA13 in clinical NPC patients and demonstrated the increased expression of HOTTIP and HOXA13 in cancer tissues, compared with those in paired normal tissues. Thus, our study suggested that the expression profile of HOTTIP and HOXA13 might be a potential biomarker for diagnosis of NPC, as well as serving as novel therapeutic targets.

In summary, we illustrated that HOTTIP promoted tumorigenesis through regulating HOXA13 expression in NPC cells, and might provide a potential therapeutic target for NPC.

## Electronic Supplementary Material

Below is the link to the electronic supplementary material.

Supplementary Material 1

## Data Availability

The datasets utilized and/or analyzed in the present study are available from the corresponding author on reasonable request.

## References

[1] Chen YP et al (2019) Nasopharyngeal carcinoma. Lancet, (394), 64–80 10.1016/S0140-6736(19)30956-0 31178151

[2] Long Z et al (2022) Trend of nasopharyngeal carcinoma mortality and years of life lost in China and its provinces from 2005 to 2020. Int J Cancer, (151), 684–691 10.1002/ijc.33998 35285029

[3] Chen YP et al (2021) Chemotherapy in Combination with Radiotherapy for definitive-intent treatment of stage II-IVA nasopharyngeal carcinoma: CSCO and ASCO Guideline. J Clin Oncol, (39), 840–859 10.1200/JCO.20.03237 33405943

[4] You R et al (2020) Efficacy and safety of Locoregional Radiotherapy with chemotherapy vs chemotherapy alone in De Novo metastatic nasopharyngeal carcinoma: a Multicenter phase 3 Randomized Clinical Trial. JAMA Oncol, (6), 1345–1352 10.1001/jamaoncol.2020.1808 PMC7378870 32701129

[5] Fei Z et al (2023) Analysis of risk characteristics for metachronous metastasis in different period of nasopharyngeal carcinoma. BMC Cancer, (23), 165 10.1186/s12885-023-10641-8 PMC9938628 36803318

[6] Kopp F, Mendell JT (2018) Functional classification and experimental dissection of long noncoding RNAs, vol 172. Cell, pp 393–407 10.1016/j.cell.2018.01.011 PMC5978744 29373828

[7] Choudhari R et al (2020) Long noncoding RNAs in cancer: from discovery to therapeutic targets. Adv Clin Chem, (95), 105–147 10.1016/bs.acc.2019.08.003 32122521

[8] Zhang W et al (2023) Knockdown of lncRNA SNHG16 attenuates the proliferation and Radioresistance of Nasopharyngeal Carcinoma cells by mediating miR-31-5p/SFN Axis. Radiat Res, (199), 124–131 10.1667/RADE-22-00163.1 36520963

[9] He SW et al (2023) .LINC00173 facilitates tumor progression by stimulating RAB1B-mediated PA2G4 and SDF4 secretion in nasopharyngeal carcinoma. Mol Oncol, (17), 518–533 10.1002/1878-0261.13375 PMC9980309 36606322

[10] Liu H et al (2023) LncRNA CASC19 Enhances the Radioresistance of Nasopharyngeal Carcinoma by Regulating the miR-340-3p/FKBP5 Axis. Int J Mol Sci, (24) 10.3390/ijms24033047 PMC9917593 36769373

[11] Li S et al (2022) ).LncRNA OIP5-AS1 knockdown targets miR-183-5p/GLUL Axis and inhibits cell proliferation, Migration and Metastasis in Nasopharyngeal Carcinoma. Front Oncol, (12), 921929 10.3389/fonc.2022.921929 PMC9214031 35756672

[12] Liang YL et al (2022) A lncRNA signature associated with tumor immune heterogeneity predicts distant metastasis in locoregionally advanced nasopharyngeal carcinoma. Nat Commun, (13), 2996 10.1038/s41467-022-30709-6 PMC9151760 35637194

[13] Wang KC, Chang HY (2011). Molecular mechanisms of long noncoding RNAs. Mol Cell.

[14] Lian Y et al (2016) HOTTIP: a critical oncogenic long non-coding RNA in human cancers. Mol Biosyst, (12), 3247–3253 10.1039/c6mb00475j 27546609

[15] Castro-Oropeza R, Melendez-Zajgla J, Maldonado V, Vazquez-Santillan K (2018) The emerging role of lncRNAs in the regulation of cancer stem cells. Cell Oncol (Dordr), (41), 585–603 10.1007/s13402-018-0406-4 PMC12995221 30218296

[16] Chen W et al (2022) Comprehensive analysis of lncRNA-mediated ceRNA networkfor hepatocellular carcinoma. Front Oncol, (12), 1042928 10.3389/fonc.2022.1042928 PMC9634570 36338699

[17] Chen X et al (2020) Exosomal Long non-coding RNA HOTTIP increases resistance of Colorectal Cancer cells to Mitomycin via Impairing MiR-214-Mediated degradation of KPNA3. Front Cell Dev Biol, (8), 582723 10.3389/fcell.2020.582723 PMC7876302 33585440

[18] Xin Y (2023). Fusobacterium nucleatum-induced exosomal HOTTIP promotes gastric cancer progression through the microRNA-885-3p/EphB2 axis. Cancer Sci.

[19] Yin F et al (2020) LncRNA HOTTIP participates in Cisplatin Resistance of Tumor cells by regulating miR-137 expression in pancreatic Cancer. Onco Targets Ther, (13), 2689–2699 10.2147/OTT.S234924 PMC7132030 32280243

[20] Yao XY et al (2021) LncRNA HOTTIP facilitates cell proliferation, invasion, and migration in osteosarcoma by interaction with PTBP1 to promote KHSRP level. Cell Cycle, (20), 283–297 10.1080/15384101.2020.1870820 PMC7889103 33475442

[21] Shen M, Li M, Liu J (2019) Long noncoding RNA HOTTIP promotes nasopharyngeal Cancer Cell Proliferation, Migration, and Invasion by inhibiting miR-4301. Med Sci Monit, (25), 778–785 10.12659/MSM.912728 PMC6360874 30685769

[22] Perez WD, Weller CR, Shou S, Stadler HS (2010) Survival of Hoxa13 homozygous mutants reveals a novel role in digit patterning and appendicular skeletal development. Dev Dyn, (239), 446–457 10.1002/dvdy.22183 PMC2981150 20034107

[23] Yokouchi Y, Sakiyama J, Kuroiwa A (1995) Coordinated expression of Abd-B subfamily genes of the HoxA cluster in the developing digestive tract of chick embryo. Dev Biol, (169), 76–89 10.1006/dbio.1995.1128 7750659

[24] Qin Z, Zhou C (2022) HOXA13 promotes gastric cancer progression partially via the FN1-mediated FAK/Src axis. Exp Hematol Oncol, (11), 7 10.1186/s40164-022-00260-7 PMC8864865 35197128

[25] Quagliata L et al (2018) High expression of HOXA13 correlates with poorly differentiated hepatocellular carcinomas and modulates sorafenib response in in vitro models. Lab Invest, (98), 95–105 10.1038/labinvest.2017.107 29035381

[26] Dong Y et al (2017) ).HOXA13 is associated with unfavorable survival and acts as a novel oncogene in prostate carcinoma. Future Oncol, (13), 1505–1516 10.2217/fon-2016-0522 28766961

[27] Han Y et al (2013) Identification and validation that up-expression of HOXA13 is a novel independent prognostic marker of a worse outcome in gastric cancer based on immunohistochemistry. Med Oncol, (30), 564 10.1007/s12032-013-0564-1 23592225

[28] Lin C et al (2017) Transcriptional and posttranscriptional regulation of HOXA13 by lncRNA HOTTIP facilitates tumorigenesis and metastasis in esophageal squamous carcinoma cells. Oncogene, (36), 5392–5406 10.1038/onc.2017.133 28534516

[29] Wu DC et al (2017) Reprogramming antagonizes the oncogenicity of HOXA13-Long noncoding RNA HOTTIP Axis in Gastric Cancer cells. Stem Cells, (35), 2115–2128 10.1002/stem.2674 28782268

[30] Feng HJ et al (2017) Silencing of FANCD2 enhances the radiosensitivity of metastatic cervical lymph node-derived head and neck squamous cell carcinoma HSC-4 cells. Int J Oncol, (50), 1241–1250 10.3892/ijo.2017.3902 28350060

[31] Xi Q et al (2015) Anticancer drugs induce hypomethylation of the acetylcholinesterase promoter via a phosphorylated-p38-DNMT1-AChE pathway in apoptotic hepatocellular carcinoma cells. Int J Biochem Cell Biol, (68), 21–32 10.1016/j.biocel.2015.08.013 26299326

[32] Wong KCW et al (2021) Nasopharyngeal carcinoma: an evolving paradigm. Nat Rev Clin Oncol, (18), 679–695 10.1038/s41571-021-00524-x 34194007

[33] Chang ET, Ye W, Zeng YX, Adami HO (2021) The evolving epidemiology of nasopharyngeal carcinoma. Cancer Epidemiol Biomarkers Prev, (30), 1035–1047 10.1158/1055-9965.EPI-20-1702 33849968

[34] Peng WX, Koirala P, Mo YY (2017) LncRNA-mediated regulation of cell signaling in cancer. Oncogene, (36), 5661–5667 10.1038/onc.2017.184 PMC6450570 28604750

[35] Wei J et al (2022) Down-regulated lncRNA ROR in tumor-educated platelets as a liquid-biopsy biomarker for nasopharyngeal carcinoma. J Cancer Res Clin Oncol. 10.1007/s00432-022-04350-1 PMC10349751 36107245

[36] Kong L et al (2018) Calycosin inhibits nasopharyngeal carcinoma cells by influencing EWSAT1 expression to regulate the TRAF6-related pathways. Biomed Pharmacother, (106), 342–348 10.1016/j.biopha.2018.06.143 29966979

[37] Scotti M, Kherdjemil Y, Roux M, Kmita M (2015) A Hoxa13:cre mouse strain for conditional gene manipulation in developing limb, hindgut, and urogenital system. Genesis, (53), 366–376 10.1002/dvg.22859 PMC4486385 25980463

[38] He YX, Song XH, Zhao ZY, Zhao H (2022). HOXA13 upregulation in gastric cancer is associated with enhanced cancer cell invasion and epithelial-to-mesenchymal transition. Eur Rev Med Pharmacol Sci.

[39] Li J, Ye M, Zhou C (2020) Expression Profile and Prognostic values of HOXA Family Members in laryngeal squamous cell Cancer. Front Oncol, (10), 368 10.3389/fonc.2020.00368 PMC7136465 32296636

[40] Chen SL The Role of the HOXA Gene Family in Acute Myeloid Leukemia., Genes et al (2019) (Basel), (10) 10.3390/genes10080621 PMC6723066 31426381

[41] Sang Y (2016). Up-regulation of long non-coding HOTTIP functions as an oncogene by regulating HOXA13 in non-small cell lung cancer. Am J Transl Res.

[42] Wong CH et al (2020) Ectopic HOTTIP expression induces noncanonical transactivation pathways to promote growth and invasiveness in pancreatic ductal adenocarcinoma. Cancer Lett, (477), 1–9 10.1016/j.canlet.2020.02.038 32120024

[43] Chang S et al (2016) HOTTIP and HOXA13 are oncogenes associated with gastric cancer progression. Oncol Rep, (35), 3577–3585 10.3892/or.2016.4743 27108607

