# Supplementary figures and images for: Long non-coding RNA HOTTIP exerts an oncogenic function by regulating HOXA13 in nasopharyngeal carcinoma
Source: Mol Biol Rep. 2023 Jul 1;50(8):6807–18. doi: 10.1007/s11033-023-08598-9 (PMC10374758; doi:10.1007/s11033-023-08598-9)

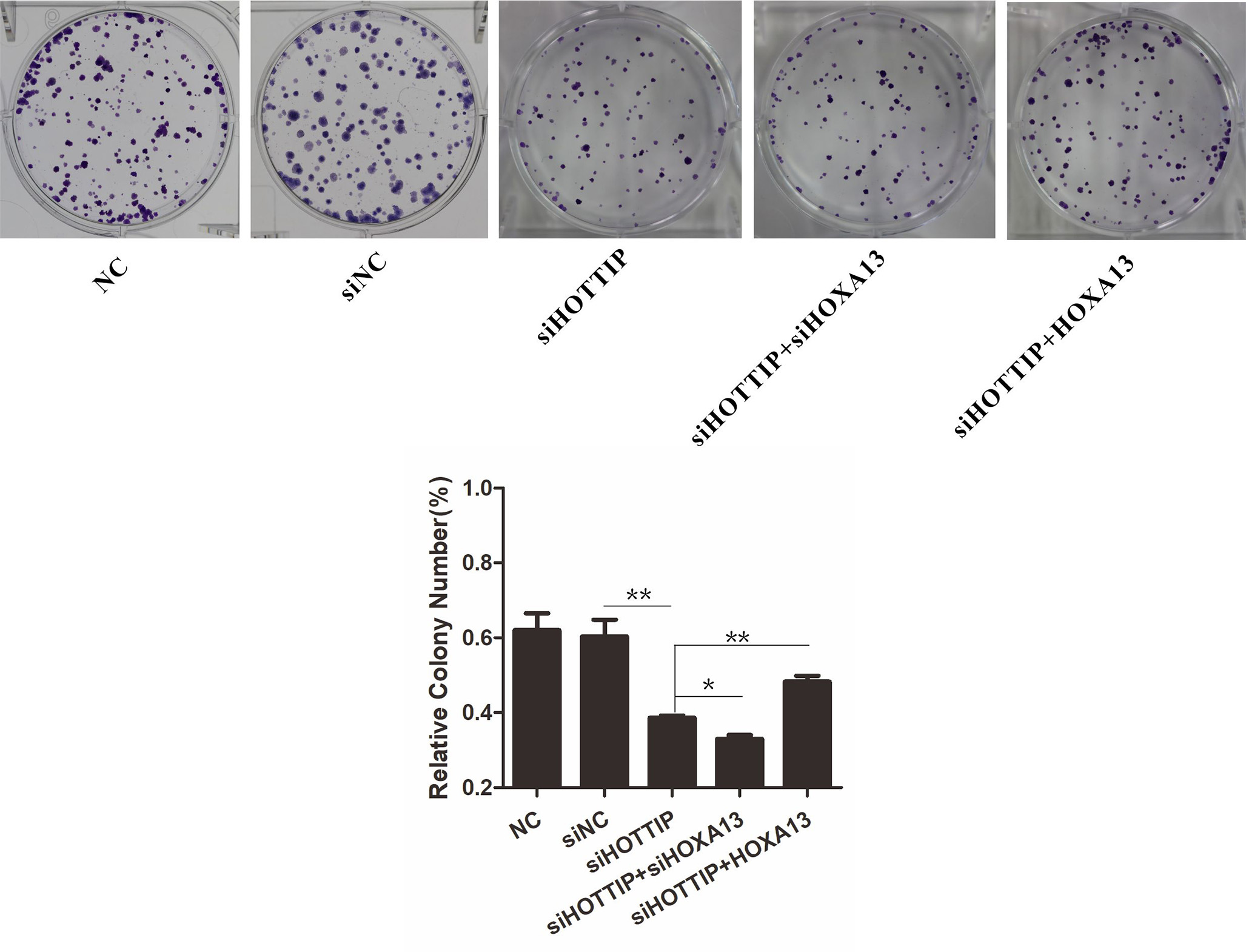

Supplement: Supplementary file 1 — Supplementary Material 1 [file 11033_2023_8598_MOESM1_ESM.jpg]
